# Supplementary material for: Mean Oral Cavity Organ-at-Risk Dose Predicts Opioid Use and Hospitalization during Radiotherapy for Patients with Head and Neck Tumors
Source: Cancers (Basel). 2024 Jan 13;16(2):349. doi: 10.3390/cancers16020349 (PMC10814074; doi:10.3390/cancers16020349)
Supplement: Supplementary file 1 [file cancers-16-00349-s001.zip › cancers-2778050-File S1.pdf]

File S1. Oral cavity OAR avoidance structure definitions and dose constraints

NRG-HN001: no definition, mean dose < 40 Gy to OC-PTV

NRG-HN002, Oral Cavity: The oral cavity will be defined as a composite structure posterior to lips consisting of the anterior ½ to 2/3 of the oral tongue/floor of mouth, buccal mucosa, and superiorly the palate, and inferiorly to the plane containing the tip of the mandible. Oral Cavity: Reduce the dose as much as possible. The mean dose should be < 32 Gy for the oral cavity. Efforts should also be made to avoid hot spots (> 60 Gy) within the non-involved oral cavity. Mean dose <=32 Gy excluding PTVs.

NRG-HN003, Cavity_Oral: For non-oral cavity cancers, the oral cavity will be defined as a composite structure consisting of the anterior 1/2 to 2/3 of the oral tongue/floor of mouth, buccal mucosa, and palate. For oral cavity cancers, the oral cavity will be defined as the subset of this composite structure that does not overlap with the PTVs. Mean dose (Dmean) <= 30 Gy. Avoid hot spots (> 60 Gy). For non-oral cavity cancers.

NRG-HN004, Lips and Oral Cavity: These should be contoured as 2 separate structures as the goal is to keep the lip dose much lower than the oral cavity dose. The definition of lips is self-explanatory. The oral cavity will be defined as a composite structure consisting of the anterior one half to two thirds of the oral tongue/floor of mouth, buccal mucosa, and palate. Mean dose < 30 Gy. D0.03cc < 60 Gy. To non-involved oral cavity.

NRG-HN005, Cavity_Oral: The oral cavity will be defined as a composite structure posterior to lips consisting of the anterior 1/2 to 2/3 of the oral tongue/floor of mouth, buccal mucosa, and superiorly the palate, and inferiorly to the plane containing the tip of the mandible (external to PTVs). Mean dose < 35 Gy to uninvolved oral cavity.

NRG-HN006, Cavity_Oral: The oral cavity will be defined as a composite structure posterior to lips consisting of the anterior 1/2 to 2/3 of the oral tongue/floor of mouth, buccal mucosa, and superiorly the palate, and inferiorly to the plane

containing the tip of the mandible (external to PTVs). Mean dose <=35 Gy. Avoid hot spots > 60 Gy. To uninvolved oral cavity.

NRG-HN008, Cavity_Oral: The oral cavity will be defined as a composite structure posterior to lips consisting of the anterior 1/2 to 2/3 of the oral tongue/floor of mouth, buccal mucosa, and superiorly the palate, and inferiorly to the plane containing the tip of the mandible (external to PTVs). Mean dose <= 35 Gy to uninvolved oral cavity.

NRG-HN009, Cavity_Oral: The oral cavity will be defined as a composite structure posterior to lips consisting of the anterior 1/2 to 2/3 of the oral tongue/floor of mouth, buccal mucosa, and superiorly the palate, and inferiorly to the plane containing the tip of the mandible (external to PTVs). Mean dose < 30 Gy to uninvolved oral cavity.

RTOG-1216, Cavity_Oral: For non-oral cavity cancers, the oral cavity will be defined as a composite structure consisting of the anterior 1/2 to 2/3 of the oral tongue/floor of mouth, buccal mucosa, and palate. For oral cavity cancers, the oral cavity will be defined as the subset of this composite structure that does not overlap with the PTVs. Mean dose <= 30 Gy, D0.03cc <= 60 Gy.

University of Michigan: The surfaces of the inner lips, buccal mucosa, tongue, base of tongue, floor of mouth, and palate were outlined on each patient's CT data set composing a distinct organ, "oral cavity" (extending to include the surface of the base of tongue). Significant associations were found between patient-reported severe dysgeusia and radiation dose to the oral cavity (*P*=.005) and tongue (*P*=.019); normal tissue complication probability for severe dysgeusia at 3 months showed mean oral cavity D_{50} doses 53 Gy and 57 Gy in the HNQOL and WUQOL questionnaires, respectively [36].

University of Toronto: Obviously, there is no single anatomic structure that would encompass all minor salivary glands. Therefore, we propose the definition of a surrogate structure, called the "Minor Oral Including Sublingual Salivary Tissue (MOIST) Target" that is intended to contain the majority of the minor salivary glands located within the mucosa covering the linings of the oral cavity and anterior

oropharynx. The MOIST Target is defined to include the minor glands of the floor of mouth, tongue, base of tongue, hard palate, soft palate, uvula, buccal mucosa, inner lips, retromolar trigone, lateral alveolar margin, anterior faucial pillar, and the sublingual glands. The sublingual glands are included as they are difficult to identify separately on cross-sectional imaging. The anterior border of the oral cavity includes the gingival reflection and the inner side of the upper and lower lips. The posterior border includes the soft palate, uvula, retromolar trigone mucosa, and more inferiorly the base of tongue. The lateral border will encompass the mandible bone and the maxilla to include the overlying gingival mucosal surfaces bilaterally and the buccal mucosa. No attempt is made to include the whole volume of the buccinator muscle. Superiorly, the "MOIST Target" includes the hard palate mucosa and mucosal reflections near the maxilla. The most superior volume of gingival mucosa is delineated up to the level of the bottom of the maxillary sinus. The inferior border is defined to include the base of tongue mucosa posteriorly and the mucosal reflection of the alveolar ridges on the mandible laterally and anteriorly. Centrally, the inferior border includes the mylohyoid and the geniohyoid muscle to include the sublingual glands. Given the differences in head position, especially when the head is in hyperextension position during the planning CT scan, the inferior border may vary slightly and include a larger part of the posterior base of tongue [25].

Affiliated Quanzhou First Hospital of Fujian Medical University, Xinyu People's Hospital, Zhejiang Cancer Hospital: Two methods, Oral Cavity Contour method- the oral mucosa was limited as follows: above to hard palate, underneath to floor of mouth, anterior to the buccal mucosa around the teeth, and posterior to tongue surface and uvula, $\geq$grade 3 toxicity V30 Gy; Mucosa Surface Contour method- the oral mucosa was defined as a 3-mm thick wall of tissue and included the following surface: buccal mucosa, buccal gingiva, gingiva proper, lingual gingiva, lingual frenulum, alveolar mucosa, labial mucosa, labial gingiva, labial frenulum, mucosal surface of the floor of the mouth, the mucosal surface of the tongue anterior to the terminal sulcus, the mucosal surface of the hard palate, and the inferior mucosal surface of the soft palate, $\geq$grade 3 toxicity V50 Gy [27].

Sacro Cuore Don Calabria Hospital, University of Palermo: The oral mucosa limits were defined as follows: hard palate superiorly, cricoid cartilage inferiorly, the buccal mucosa around the teeth anteriorly, and the posterior pharyngeal wall posteriorly. Mucositis ≥G2 was found to be statistically related to chemotherapy, weight loss, dysphagia ≥G2, total oral mucosa D_{mean} ≥50 Gy and D_{max} ≥65 Gy, V_{45} Gy >40%, V_{50} Gy >30%, and V_{55} Gy >20% of the oral mucosa minus target PTVs. A ratio between total oral mucosa and oral mucosa minus target PTVs >2.5 is related to G3 mucositis (p =.03) [54].

The Institute of Cancer Research and The Royal Marsden NHS Foundation Trust: The mucosal surface contours were defined as a 3 mm thick wall of tissue. The outlined mucosal surface contours included the following surfaces: buccal mucosa, buccal gingiva, gingiva proper, lingual gingiva, lingual frenulum, alveolar mucosa, labial mucosa, labial gingiva, labial frenulum, mucosal surface of the floor of mouth, mucosal surface of the tongue anterior to the terminal sulcus, and the mucosal surface of the hard palate. The superior extent was defined to be the superior border of the labial mucosa of the upper lip anteriorly, the roof of the palate posteriorly and the superior extent of the buccal mucosa laterally. The inferior extent was formed by the inferior border of the labial mucosa of the lower lip anteriorly, the surface of the tongue posteriorly and the inferior extents of the floor of mouth mucosa and buccal mucosa laterally. The lateral extents of the buccal mucosa formed the lateral borders. The anterior border followed the alveolar mucosa, and the posterior extent of the hard palate formed the posterior border. The mucosal surface contours were initially delineated as a single line and once complete, expanded to a 3 mm annulus [29].

International Consensus OAR Contouring Guidelines: The delineation of the extended oral cavity was based partly on Hoebers et al. For the sake of simplicity and consistency, the extended oral cavity structure was defined posterior to the internal arch of the mandible and maxilla. The mucosa anterior to the mandible and maxilla is included in the contour of the lips, and the mucosa lateral to the mandible and maxilla is included in the buccal mucosa. Anatomic boundaries: Cranial-Hard palate mucosa and mucosal reflections near the maxilla, Caudal-The base of tongue, mucosa and hyoid, posteriorly, and the mylohyoid muscle and

anterior belly of the digastric muscle, anteriorly, Anterior-Inner surface of the mandible and maxilla, Posterior-Posterior borders of soft palate, uvula, and more inferiorly the base of tongue, Lateral-Inner surface of the mandible and maxilla. Posterior to mandible and maxilla, no inner surface of the lips.

For research purposes, the extended oral cavity can be subdivided into oral tongue and anterior oropharynx, by drawing a vertical line from the posterior hard palate to the hyoid (circumvallate line) [26].

Chang Gung Memorial Hospital: The surfaces of the inner lips, buccal mucosa, tongue, base of the tongue, floor of the mouth, and palate were outlined on each computed tomography (CT) data set to form a distinct organ, the "oral cavity" (extended to include the surface of the base of the tongue). Positive correlation occurred between objectively measured taste loss for the 4 taste qualities and subjective perception of taste loss. Only oral cavity mean dose 4000 cGy or greater predicted taste dysfunction 3 months after RT. The mean oral cavity doses to the predicted 15%(D_{15}), 25%(D_{25}), and 50% (D_{50}) probabilities were 25, 38, and 60 Gy at 3 months and 57, 60, and 64 Gy at 6 months, respectively [32].

University of North Carolina: The OARs were generally segmented as described in a publication by Brouwer et al. (International Consensus OAR Contouring Guidelines outlined above). The anterior two-thirds of the tongue were defined as the oral tongue, and the posterior third as the base of tongue. The region inferior and anterior to the tongue was delineated as the floor of mouth. Nineteen total structures were contoured including the left and right parotid glands, left and right submandibular glands, left and right sublingual glands, oral tongue, base of tongue, floor of mouth, soft and hard palates, buccal mucosa, upper and lower lips. The only significant factor related to dysgeusia at 12 months was mean dose to the oral cavity (P = .009). On examining substructures, the mean dose to the floor of mouth was implicated for the dose relationship to 6-month xerostomia (P = .04), and the oral tongue was found to be implicated for the relationship for 12-month dysgeusia (P = .04) [31].

The University of Hong Kong-Shenzhen Hospital, National Cancer Center Singapore, Memorial Sloan-Kettering Cancer Center, State Key Laboratory of

Oncology in South China, Sun Yat-sen University Cancer Center: Tongue/Oral Cavity, Cranial-Post. edge of the hard palate or soft palate, Caudal- Disappearance of anterior belly of digastric muscle, Anterior-Post. edge of mandible or is free, Posterior- Palate, oropharynx, the palatine tonsil, hyoid bone, Lateral- Med. edge of the mandible or inferior alveoli socket [28].
