# Supplementary material for: Mean Oral Cavity Organ-at-Risk Dose Predicts Opioid Use and Hospitalization during Radiotherapy for Patients with Head and Neck Tumors
Source: Cancers (Basel). 2024 Jan 13;16(2):349. doi: 10.3390/cancers16020349 (PMC10814074; doi:10.3390/cancers16020349)
Supplement: Supplementary file 1 [file cancers-16-00349-s001.zip › cancers-2778050-Table S1.pdf]

Table S1. International Classification of Diseases (ICD)-10 codes used in the database search.

ICD-10 code
C01
C02.2, C02.3
C03, C03.1, C03.9
C04, C04.1, C04.9
C05, C05.1
C06, C06.2
C07
C08
C09, C09.9
C10.2, C10.8, C10.9
C11.2, C11.9
C13
C30
C31, C31.1, C31.2, C31.3, C31.9
C32, C32.1
C33
C41
C43.4
C44.111, C44.222, C44.229, C44.319, C44.320, C44.329, C44.42, C44.92, C44.99, C44.519
C49.9
C69.01, C69.51, C69.52
C73
C75.5
C77, C77.9
C79.49
C80.1
C4A.31, C4A.39
D02
D16.5
D35.6
