# Supplementary material for: Mean Oral Cavity Organ-at-Risk Dose Predicts Opioid Use and Hospitalization during Radiotherapy for Patients with Head and Neck Tumors
Source: Cancers (Basel). 2024 Jan 13;16(2):349. doi: 10.3390/cancers16020349 (PMC10814074; doi:10.3390/cancers16020349)
Supplement: Supplementary file 1 [file cancers-16-00349-s001.zip › cancers-2778050-Table S2.pdf]

Table S2. Univariable Analysis-Variables Associated with Toxicity

Toxicity	Time Point	n	Variable	OR (95% CI)	p-value
CTCAE v4.03 dehydration >grade 1	End of treatment	57/194	Cytotoxic chemotherapy	7.15 (3.50,14.63)	<.0001
			Oral cavity OAR Dmax dose	1.55(1.12,2.14)	0.0083
			Oral cavity OAR mean dose	1.45(1.15,1.82)	0.0015
			Oral cavity OAR V10	1.51(1.11,2.07)	0.0093
			Oral cavity OAR V20	1.62(1.15,2.28)	0.0061
			Oral cavity OAR V30	1.66(1.12,2.46)	0.0116
			Oral cavity OAR V40	1.60(1.03,2.48)	0.0350
			Oral cavity OAR V60	1.84(1.01,3.37)	0.0468
			Oral cavity OAR V70	5.71(1.28,25.56)	0.0227
			Mean left parotid dose	1.28(1.07,1.53)	0.0069
			Mean right parotid dose	1.39(1.14,1.69)	0.0012
			Mean total parotid dose	1.39(1.14,1.69)	<0.0001
			Mean left SMG dose	1.26(1.10,1.44)	0.0009
			Mean right SMG dose	1.29(1.12,1.47)	0.0003
			Protons	2.73(1.43,5.22)	0.0024
CTCAE v4.03 dry mouth >grade 1	End of treatment	40/193	Oral cavity OAR Dmax dose	1.5(1.03,2.18)	0.0336
			Oral cavity OAR mean dose	1.58(1.23,2.03)	0.0004
			Oral cavity OAR V10	1.82(1.27,2.62)	0.0011
			Oral cavity OAR V20	1.99(1.36,2.92)	0.0004
			Oral cavity OAR V30	1.9(1.25,2.89	0.0028
			Oral cavity OAR V40	1.85(1.17,2.93)	0.0082
			Oral cavity OAR V50	1.86(1.11,3.10)	0.0183
			Mean left parotid dose	1.31(1.07,1.59)	0.0073
			Mean total parotid dose	1.33(1.04,1.70)	0.0206
			Mean left SMG dose	1.3(1.10,1.53)	0.0020
			Mean right SMG dose	1.24(1.06,1.46)	0.0063
CTCAE v4.03 dry mouth >grade 1	3 months	25/188	Oral cavity OAR mean dose	1.64(1.22,2.19)	0.0010
			Oral cavity OAR V10	1.62(1.06,2.47)	0.0243
			Oral cavity OAR V20	1.87(1.20,2.90)	0.0058
			Oral cavity OAR V30	2.01(1.24,3.25)	0.0045
			Oral cavity OAR V40	2.07(1.23,3.48)	0.0061
			Oral cavity OAR V50	2.12(1.19,3.77)	0.0109
			Mean left parotid dose	1.47(1.17,1.85)	0.0008
			Mean right parotid dose	1.33(1.04,1.71)	0.0240
			Mean total parotid dose	1.77(1.29,2.44)	0.0004
			Mean left SMG dose	1.67(1.23,2.28	0.0011
			Mean right SMG dose	1.28(1.04,1.56)	0.0185
CTCAE v4.03 dry mouth >grade 1	6 months	15/179	Cytotoxic chemotherapy	3.69(1.13,12.08)	0.0308
			Oral cavity OAR mean dose	1.89(1.28,2.79)	0.0013
			Oral cavity OAR V10	1.83(1.07,3.12)	0.0261
			Oral cavity OAR V20	2.24(1.29,3.90)	0.0044
			Oral cavity OAR V30	2.47(1.36,4.47)	0.0028
			Oral cavity OAR V40	2.57(1.36,4.85)	0.0037
			Oral cavity OAR V50	2.69(1.33,5.43)	0.0059
			Oral cavity OAR V60	2.9(1.26,6.71)	0.0126
			Mean left parotid dose	1.62(1.20,2.19)	0.0017
			Mean right parotid dose	1.7(1.23,2.35)	0.0013

			Mean total parotid dose	2.39(1.53,3.73)	0.0001
			Mean left SMG dose	1.85(1.18,2.91)	0.0071
			Mean right SMG dose	1.53(1.08,2.17)	0.0169
CTCAE v4.03 dry mouth >grade 1	1 year	10/175	Cytotoxic chemotherapy	5.57(1.15,27.02)	0.0332
			Oral cavity OAR Dmax dose	2.89(1.02,8.16)	0.0458
			Oral cavity OAR mean dose	2.53(1.51,4.23)	0.0004
			Oral cavity OAR V20	2.49(1.28,4.87)	0.0075
			Oral cavity OAR V30	2.96(1.46,6.01)	0.0027
			Oral cavity OAR V40	3.19(1.51,6.71)	0.0023
			Oral cavity OAR V50	3.5(1.56,7.86)	0.0023
			Oral cavity OAR V60	4.5(1.75,11.54)	0.0018
			Mean left parotid dose	1.48(1.06,2.06)	0.0208
			Mean right parotid dose	1.78(1.20,2.65)	0.0044
			Mean total parotid dose	2.3(1.39,3.82)	0.0012
			Mean left SMG dose	1.99(1.04,3.80)	0.0364
			Protons	4.54(1.22,16.87)	0.0240
CTCAE v4.03 dry mouth >grade 1	2 years	9/157	Oral cavity OAR mean dose	1.94(1.19,3.16)	0.0075
			Oral cavity OAR V30	2.16(1.05,4.46)	0.0374
			Oral cavity OAR V40	2.37(1.11,5.10)	0.0266
			Oral cavity OAR V50	2.66(1.15,6.17)	0.0226
			Oral cavity OAR V60	3.36(1.21,9.32)	0.0197
			Mean right parotid dose	1.51(1.01,2.27)	0.0453
			Mean total parotid dose	1.81(1.13,2.90)	0.0132
			Protons	5.59(1.33,23.44)	0.0186
CTCAE v4.03 dysgeusia >grade 1	End of Treatment	40/191	Oral cavity OAR Dmax dose	1.5(1.04,2.17)	0.0305
			Oral cavity OAR mean dose	1.51(1.18,1.94)	0.0013
			Oral cavity OAR V10	2.04(1.40,2.95)	0.0002
			Oral cavity OAR V20	2.11(1.42,3.11)	0.0002
			Oral cavity OAR V30	2.1(1.36,3.25)	0.0008
			Oral cavity OAR V40	2.02(1.25,3.24)	0.0038
			Oral cavity OAR V50	2.04(1.19,3.49)	0.0093
			Oral cavity OAR V60	2.35(1.23,4.49)	0.0096
			Oral cavity OAR V70	6.21(1.44,26.87)	0.0145
			Mean right parotid dose	1.42(1.13,1.79)	0.0024
			Mean total parotid dose	1.34(1.05,1.71)	0.0169
			Mean right SMG dose	1.39(1.17,1.66)	0.0002
CTCAE v4.03 dysgeusia >grade 1	3 months	15/186	Oral cavity OAR Dmax dose	2.99(1.26,7.10)	0.0132
			Oral cavity OAR mean dose	1.76(1.22,2.53)	0.0024
			Oral cavity OAR V10	1.98(1.15,3.39)	0.0134
			Oral cavity OAR V20	2.43(1.38,4.26)	0.0020
			Oral cavity OAR V30	2.45(1.35,4.46)	0.0033
			Oral cavity OAR V40	2.32(1.22,4.41)	0.0101
			Oral cavity OAR V50	2.34(1.15,4.78)	0.0196
			Oral cavity OAR V60	2.56(1.11,5.92)	0.0279
			Oral cavity OAR V70	8.75(1.87,40.87)	0.0058
			Mean left parotid dose	1.42(1.08,1.86)	0.0119
			Mean right parotid dose	1.4(1.02,1.91)	0.0367
			Mean total parotid dose	1.79(1.22,2.63)	0.0028
			Mean left SMG dose	1.43(1.06,1.92)	0.0191
			Mean right SMG dose	1.45(1.08,1.95)	0.0124

CTCAE v4.03 dysphagia >grade 1	End of Treatment	53/185	Cytotoxic chemotherapy	2.17(1.13,4.15)	0.0193
			Oral cavity OAR mean dose	1.5(1.18,1.90)	0.0011
			Oral cavity OAR V10	1.98(1.40,2.79)	0.0001
			Oral cavity OAR V20	1.84(1.27,2.66)	0.0011
			Oral cavity OAR V30	1.81(1.19,2.77)	0.0058
			Oral cavity OAR V40	1.84(1.15,2.96)	0.0115
			Oral cavity OAR V50	1.94(1.13,3.33)	0.0165
			Oral cavity OAR V60	2.29(1.18,4.43)	0.0137
			Mean right parotid dose	1.38(1.12,1.69)	0.0021
			Mean total parotid dose	1.47(1.16,1.86)	0.0014
			Mean left SMG dose	1.41(1.20,1.65)	<.0001
			Mean right SMG dose	1.45(1.22,1.71)	<.0001
			Protons	3.38(1.73,6.63)	0.0004
CTCAE v4.03 dysphagia >grade 1	3 months	34/181	Oral cavity OAR mean dose	1.7(1.29,2.26)	0.0002
			Oral cavity OAR V10	1.93(1.30,2.87)	0.0011
			Oral cavity OAR V20	1.98(1.30,3.02)	0.0015
			Oral cavity OAR V30	2.07(1.29,3.33)	0.0026
			Oral cavity OAR V40	2.13(1.26,3.62)	0.0049
			Oral cavity OAR V50	2.2(1.21,3.99)	0.0096
			Oral cavity OAR V60	2.37(1.17,4.79)	0.0161
			Mean left parotid dose	1.24(1.01,1.52)	0.0428
			Mean right parotid dose	1.39(1.11,1.75)	0.0047
			Mean total parotid dose	1.58(1.20,2.08)	0.0012
			Mean left SMG dose	1.46(1.18,1.81)	0.0006
			Mean right SMG dose	1.45(1.17,1.80)	0.0007
			Protons	3.47(1.60,7.5)	0.0016
CTCAE v4.03 dysphagia >grade 1	6 months	23/174	Oral cavity OAR Dmax dose	2(1.08,3.70)	0.0268
			Oral cavity OAR mean dose	2.17(1.50,3.13)	<.0001
			Oral cavity OAR V10	2.05(1.28,3.27)	0.0026
			Oral cavity OAR V20	2.27(1.38,3.73)	0.0013
			Oral cavity OAR V30	2.69(1.52,4.76)	0.0007
			Oral cavity OAR V40	2.96(1.56,5.61)	0.0009
			Oral cavity OAR V50	3.3(1.60,6.80)	0.0012
			Oral cavity OAR V60	4.18(1.78,9.80)	0.0010
			Oral cavity OAR V70	13.79(2.10,90.70)	0.0063
			Mean right parotid dose	1.48(1.13,1.93)	0.0047
			Mean total parotid dose	1.63(1.18,2.25)	0.0029
			Mean left SMG dose	1.57(1.18,2.10)	0.0022
			Mean right SMG dose	1.68(1.20,2.36)	0.0024
			Protons	2.63(1.07,6.45)	0.0342
CTCAE v4.03 dysphagia >grade 1	1 year	18/169	Age	1.81(1.14,2.88)	0.0123
			Hypertension	2.74(1.01,7.49)	0.0489
			Oral cavity OAR mean dose	1.73(1.19,2.51)	0.0039
			Oral cavity OAR V10	1.77(1.07,2.93)	0.0274
			Oral cavity OAR V20	1.88(1.09,3.25)	0.0228
			Oral cavity OAR V30	2.09(1.12,3.91)	0.0210
			Oral cavity OAR V40	2.18(1.08,4.38)	0.0293
			Oral cavity OAR V50	2.37(1.08,5.19)	0.0311
			Oral cavity OAR V60	3.09(1.24,7.70)	0.0154
			Oral cavity OAR V70	16.25(2.32,113.56)	0.0050

			Mean left parotid dose	1.42(1.09,1.85)	0.0094
			Mean total parotid dose	1.47(1.05,2.06)	0.0262
			Mean left SMG dose	1.71(1.18,2.47)	0.0044
			Mean right SMG dose	1.35(1.06,1.73)	0.0162
CTCAE v4.03 dysphagia >grade 1	2 years	16/153	Oral cavity OAR mean dose	1.54(1.04,2.29)	0.0311
			Mean left parotid dose	1.33(1.02,1.75)	0.0382
			Mean total parotid dose	1.56(1.08,2.25)	0.0172
			Mean left SMG dose	1.54(1.10,2.16)	0.0125
			Mean right SMG dose	1.36(1.04,1.79)	0.0267
			Protons	3.89(1.35,11.25)	0.0120
CTCAE v4.03 oral pain >grade 1	End of Treatment	27/191	Oral cavity OAR mean dose	1.82(1.35,2.45)	<.0001
			Oral cavity OAR V10	1.86(1.23,2.82)	0.0034
			Oral cavity OAR V20	2.09(1.36,3.23)	0.0008
			Oral cavity OAR V30	2.31(1.43,3.73)	0.0006
			Oral cavity OAR V40	2.55(1.51,4.31)	0.0004
			Oral cavity OAR V50	2.92(1.62,5.25)	0.0003
			Oral cavity OAR V60	3.57(1.78,7.15)	0.0003
			Protons	3.21(1.40,7.37)	0.0061
CTCAE v4.03 salivary duct inflammation >grade 1	End of Treatment	51/192	Cytotoxic chemotherapy	3.63(1.84,7.19)	0.0002
			Oral cavity OAR Dmax dose	1.57(1.11,2.21)	0.0106
			Oral cavity OAR mean dose	1.96(1.50,2.55)	<.0001
			Oral cavity OAR V10	2.19(1.54,3.13)	<.0001
			Oral cavity OAR V20	2.19(1.50,3.19)	<.0001
			Oral cavity OAR V30	2.26(1.48,3.46)	0.0002
			Oral cavity OAR V40	2.29(1.43,3.65)	0.0005
			Oral cavity OAR V50	2.44(1.44,4.15)	0.0010
			Oral cavity OAR V60	3.25(1.68,6.31)	0.0005
			Oral cavity OAR V70	17.4(2.59,116.84)	0.0033
			Mean left parotid dose	1.52(1.25,1.85)	<.0001
			Mean right parotid dose	1.46(1.19,1.80)	0.0003
			Mean total parotid dose	1.98(1.51,2.59)	<.0001
			Mean left SMG dose	1.69(1.37,2.08)	<.0001
			Mean right SMG dose	1.69(1.38,2.08)	<.0001
			Protons	3.15(1.61,6.15)	0.0006
CTCAE v4.03 salivary duct inflammation >grade 1	3 months	13/187	Oral cavity OAR mean dose	1.87(1.26,2.78)	0.0020
			Oral cavity OAR V20	1.96(1.09,3.52)	0.0243
			Oral cavity OAR V30	2.27(1.20,4.27)	0.0113
			Oral cavity OAR V40	2.41(1.22,4.76)	0.0110
			Oral cavity OAR V50	2.59(1.22,5.50)	0.0130
			Oral cavity OAR V60	2.98(1.25,7.13)	0.0140
			Oral cavity OAR V70	5.37(1.13,25.62)	0.0350
			Mean left parotid dose	1.56(1.15,2.11)	0.0043
			Mean right parotid dose	1.48(1.07,2.04)	0.0183
			Mean total parotid dose	2.05(1.33,3.14)	0.0011
			Mean left SMG dose	2.94(1.23,7.02)	0.0150
			Mean right SMG dose	1.53(1.07,2.19)	0.0208
Measured weight loss ≥10%	End of Treatment	21/186	Cytotoxic chemotherapy	5.63(1.81,17.45)	0.0028
			Oral cavity OAR mean dose	1.51(1.11,2.05)	0.0094
			Oral cavity OAR V10	1.98(1.24,3.16)	0.0041

			Oral cavity OAR V20	1.87(1.17,2.98)	0.0089
			Oral cavity OAR V30	1.86(1.12,3.09)	0.0173
			Oral cavity OAR V70	4.53(1.07,19.14)	0.0400
			Mean left parotid dose	1.45(1.13,1.85)	0.0033
			Mean right parotid dose	1.37(1.05,1.78)	0.0206
			Mean total parotid dose	1.82(1.29,2.57)	0.0007
			Mean left SMG dose	1.35(1.05,1.74)	0.0191
			Mean right SMG dose	1.4(1.11,1.77)	0.0051
			Protons	3.56(1.40,9.01)	0.0075
Measured weight loss ≥10%	3 months	55/182	Cytotoxic chemotherapy	2.41(1.26,4.62)	0.0079
			Oral cavity OAR Dmax dose	1.88(1.27,2.79)	0.0017
			Oral cavity OAR mean dose	1.47(1.16,1.86)	0.0012
			Oral cavity OAR V10	1.76(1.26,2.45)	0.0009
			Oral cavity OAR V20	1.88(1.31,2.69)	0.0007
			Oral cavity OAR V30	1.93(1.29,2.89)	0.0014
			Oral cavity OAR V40	1.86(1.20,2.90)	0.0058
			Oral cavity OAR V50	1.8(1.09,2.96)	0.0217
			Oral cavity OAR V70	4.38(1.04,18.39)	0.0435
			Mean left parotid dose	1.36(1.13,1.64)	0.0009
			Mean right parotid dose	1.43(1.16,1.75	0.0007
			Mean total parotid dose	1.72(1.34,2.20)	<.0001
			Mean left SMG dose	1.23(1.07,1.41)	0.0032
			Mean right SMG dose	1.3(01.13,1.49)	0.0003
Measured weight loss ≥10%	6 months	48/159	Cytotoxic chemotherapy	4.31(2.07,8.97)	<.0001
			Oral cavity OAR Dmax dose	1.98(1.26,3.13)	0.0033
			Oral cavity OAR mean dose	1.48(1.14,1.93)	0.0033
			Oral cavity OAR V10	1.68(1.17,2.42)	0.0049
			Oral cavity OAR V20	1.85(1.24,2.76)	0.0026
			Oral cavity OAR V30	1.79(1.15,2.77)	0.0098
			Oral cavity OAR V40	1.69(1.04,2.72)	0.0327
			Mean left parotid dose	1.67(1.32,2.10)	<.0001
			Mean right parotid dose	1.51(1.19,1.92)	0.0007
			Mean total parotid dose	2.13(1.56,2.91)	<.0001
			Mean left SMG dose	1.42(1.19,1.69)	<.0001
			Mean right SMG dose	1.45(1.22,1.73)	<.0001
Measured weight loss ≥10%	1 year	47/159	Cytotoxic chemotherapy	3.63(1.77,7.43)	0.0004
			Oral cavity OAR Dmax dose	2.09(1.29,3.38)	0.0027
			Oral cavity OAR mean dose	1.90(1.41,2.56)	0.0027
			Oral cavity OAR V10	1.74(1.21,2.50)	0.0028
			Oral cavity OAR V20	2.19(1.44,3.34)	0.0003
			Oral cavity OAR V30	2.17(1.35,3.47)	0.0014
			Oral cavity OAR V40	2.06(1.23,3.43)	0.0060
			Oral cavity OAR V50	1.91(1.08,3.39)	0.0266
			Oral cavity OAR V70	33.19(2.57,428.80)	0.0073
			Mean left parotid dose	1.59(1.27,1.98)	<.0001
			Mean right parotid dose	1.56(1.23,1.98)	0.0003
			Mean total parotid dose	2.05(1.52,2.76)	<.0001
			Mean left SMG dose	1.46(1.22,1.75)	<.0001
			Mean right SMG dose	1.48(1.24,1.77)	<.0001
Measured weight loss ≥10%	2 years	34/148	Oral cavity OAR Dmax dose	1.6(1.03,2.48)	0.0363

			Oral cavity OAR mean dose	1.45(1.08,1.94)	0.0130
			Oral cavity OAR V20	1.61(1.04,2.50)	0.0322
			Mean left parotid dose	1.32(1.06,1.64)	0.0131
			Mean right parotid dose	1.28(1.00,1.64)	0.0473
			Mean total parotid dose	1.51(1.14,2.00)	0.0036
			Mean left SMG dose	1.31(1.09,1.59)	0.0045
			Mean right SMG dose	1.28(1.07,1.53)	0.0078
PROMIS 10 global physical health T-score decrease ≥10 points	End of Treatment	20/79	Cytotoxic chemotherapy	4.55(1.52,13.64)	0.0068
PROMIS 10 global physical health T-score decrease ≥10 points	3 months	12/84	Oral cavity OAR Dmax dose	5.22(1.66,16.44)	0.0048
PROMIS 10 global physical health T-score decrease ≥10 points	1 year	10/83	Age	2.19(1.07,4.46)	0.0311
			Ever Smoker	8.76(1.05,72.69)	0.0445
PROMIS 10 global mental health T-score decrease ≥10 points	1 year	9/83	Oral cavity OAR V10	0.23(0.08,0.71)	0.0105
			Oral cavity OAR V20	0.19(0.05,0.74)	0.0166
			Oral cavity OAR V30	0.18(0.04,0.88)	0.0348
			Mean total parotid dose	0.53(0.30,0.93)	0.0265
			Mean left SMG dose	0.74(0.57,0.96)	0.0245
EORTC pain decrease ≥ 10 pts	End of Treatment	43/70	Mean left parotid dose	1.39(1.00,1.94)	0.0477
EORTC pain decrease ≥ 10 pts	3 months	23/75	Male	0.27(0.09,0.81)	0.0197
EORTC pain decrease ≥ 10 pts	1 year	16/76	Age	1.75(1.06,2.89)	0.0299
EORTC swallow decrease ≥ 10 pts	End of Treatment	40/72	Mean left SMG dose	1.26(1.03,1.53)	0.0235
			Mean right SMG dose	1.21(1.00,1.47)	0.0472
EORTC swallow decrease ≥ 10 pts	3 months	18/75	Oral cavity OAR Dmax dose	2.94(1.21,7.15)	0.0171
			Mean right parotid dose	1.73(1.13,2.64)	0.0110
			Mean total parotid dose	1.74(1.11,2.75)	0.0167
			Mean left SMG dose	1.62(1.14,2.32)	0.0076
			Mean right SMG dose	1.46(1.10,1.94)	0.0082
EORTC swallow decrease ≥ 10 pts	6 months	13/75	Oral cavity OAR Dmax dose	2.45(1.04,5.78)	0.0405
			Mean total parotid dose	1.71(1.02,2.87)	0.0424
			Mean left SMG dose	1.56(1.07,2.26)	0.0193
			Mean right SMG dose	1.57(1.10,2.24)	0.0129
EORTC swallow decrease ≥ 10 pts	2 years	16/64	Age	1.98(1.11,3.55)	0.0212
			Oral cavity OAR V70	229.8(3.48,>9999.99)	0.0110
			Mean right SMG dose	1.35(1.04,1.75)	0.0222
EORTC saliva decrease ≥ 10 pts	End of Treatment	49/71	Male	3.7(1.22,11.21)	0.0205
			Oral cavity OAR mean dose	1.59(1.03,2.47)	0.0379
			Oral cavity OAR V10	1.93(1.07,3.49)	0.0295
			Oral cavity OAR V20	2.26(1.07,4.77)	0.0320
			Oral cavity OAR V30	2.42(1.03,5.72)	0.0435
			Oral cavity OAR V40	2.68(1.03,6.98)	0.0430
			Mean right SMG dose	1.29(1.03,1.62)	0.0264
EORTC saliva decrease ≥ 10 pts	6 months	45/72	Ever smoker	2.75(1.03,7.38)	0.0440
			Mean left parotid dose	1.51(1.08,2.10)	0.0149
EORTC saliva decrease ≥ 10 pts	1 year	37/76	Mean right parotid dose	1.41(1.03,1.93)	0.0336

			Mean right SMG dose	1.24(1.02,1.49)	0.0278
EORTC saliva decrease ≥ 10 pts	2 years	31/65	Mean right SMG dose	1.25(1.02,1.53)	0.0321
EORTC senses decrease ≥ 10 pts	End of Treatment	54/69	Oral cavity OAR mean dose	1.78(1.04,3.05)	0.0355
			Mean right SMG dose	1.34(1.03,1.75)	0.0276
EORTC senses decrease ≥ 10 pts	3 months	49/74	Age	1.84(1.25,2.70)	0.0021
			Hypertension	3.54(1.14,10.95)	0.0283
			Mean left parotid dose	1.38(1.01,1.90)	0.0439
EORTC senses decrease ≥ pts	6 months	41/73	Age	1.52(1.07,2.15)	0.0183
			Oral cavity Dmax dose	1.62(1.08,2.44)	0.0207
EORTC senses decrease ≥ 10 pts	1 year	39/75	Age	1.9(1.27,2.85)	0.0018
EORTC senses decrease ≥ 10 pts	2 years	29/61	Age	3.61(1.72,7.55)	0.0007
EORTC speech decrease ≥ 10 pts	3 months	20/73	Male	0.25(0.08,0.78)	0.0168
EORTC speech decrease ≥ 10 pts	6 months	20/75	Male	0.18(0.06,0.56)	0.0028
EORTC speech decrease ≥ 10 pts	2 years	19/65	Oral cavity OAR Dmax dose	2.35(1.01,5.43)	0.0463
EORTC social eating decrease ≥ 10 pts	End of Treatment	46/71	Oral cavity OAR mean dose	1.52(1.01,2.31)	0.0467
			Oral cavity OAR V10	1.84(1.04,3.23)	0.0355
EORTC social eating decrease ≥ 10 pts	3 months	29/74	Oral cavity OAR Dmax dose	2.59(1.24,5.43)	0.0117
			Mean total parotid dose	1.46(1.02,2.11)	0.0396
			Mean left SMG dose	1.3(1.04,1.62)	0.0219
EORTC social eating decrease ≥ 10 pts	6 months	14/75	Age	2.39(1.25,4.56)	0.0085
			Oral cavity OAR Dmax dose	2.45(1.05,5.72)	0.0389
EORTC social eating decrease ≥ 10 pts	1 year	22/77	Ever smoker	3.53(1.14,10.90)	0.0286
			Oral cavity OAR Dmax dose	2.69(1.13,6.42)	0.0253
EORTC social eating decrease ≥ 10 pts	2 years	17/65	Age	2.35(1.25,4.40)	0.0077
EORTC feeding tube	End of Treatment	10/75	Oral cavity OAR mean dose	2.14(1.27,3.60)	0.0041
			Oral cavity OAR V10	2.19(1.07,4.48)	0.0313
			Oral cavity OAR V20	2.76(1.30,5.90)	0.0086
			Oral cavity OAR V30	3.35(1.43,7.85)	0.0054
			Oral cavity OAR V40	3.69(1.46,9.37)	0.0059
			Oral cavity OAR V50	3.94(1.44,10.75)	0.0074
			Oral cavity OAR V60	3.93(1.34,11.53)	0.0126
			Oral cavity OAR V70	18.73(1.26,277.98)	0.0333
			Mean right parotid dose	2.05(1.25,3.37)	0.0043
			Mean total parotid dose	1.88(1.08,3.28)	0.0260
			Mean right SMG dose	1.69(1.07,2.65)	0.0236
EORTC weight loss	End of treatment	14/75	Mean left SMG dose	1.32(1.01,1.72)	0.0405
EORTC weight loss	3 months	10/75	Cytotoxic chemotherapy	5.63(1.11,28.62)	0.0373
Start of opioid pain medication use	During or within 30 days of completing RT	79/122	DM prior to RT	0.2 (0.05, 0.83)	0.0266
			Cytotoxic chemotherapy	4.97 (2.05, 12.05)	0.0004
			Oral cavity OAR mean dose	1.71 (1.22, 2.39)	0.0019
			Oral cavity OAR V10	1.89 (1.22, 2.94)	0.0043
			Oral cavity OAR V20	1.85 (1.13, 3.05)	0.0150
			Oral cavity OAR V30	1.91 (1.08, 3.38)	0.0270
			Oral cavity OAR V40	1.95 (1.02, 3.73)	0.0438

			Mean left parotid dose	1.39 (1.07, 1.81)	0.0125
			Mean right parotid dose	1.38 (1.03, 1.85)	0.0288
			Mean total parotid dose	1.66 (1.22, 2.26)	0.0013
			Mean left SMG dose	1.43 (1.20, 1.69)	<0.0001
			Mean right SMG dose	1.25 (1.08, 1.45)	0.0035
Hospitalization	During or within 30 days of completing RT	40/196	Cytotoxic chemotherapy	2.74 (1.33, 5.66)	0.0063
			Oral cavity OAR mean dose	1.38 (1.08, 1.76)	0.0093
			Oral cavity OAR V10	1.86 (1.30, 2.67)	0.0007
			Oral cavity OAR V20	1.80 (1.24, 2.61)	0.0021
			Oral cavity OAR V30	1.79 (1.18, 2.71)	0.0062
			Oral cavity OAR V40	1.70 (1.08, 2.67)	0.0225
			Oral cavity OAR V50	1.72 (1.03, 2.87)	0.0377
			Oral cavity OAR V60	1.86 (1.00, 3.45)	0.0483
			Oral cavity OAR V70	6.83 (1.55, 30.13)	0.0111
			Mean right parotid dose	1.24 (1.00, 1.53)	0.0498
			Mean total parotid dose	1.31 (1.03, 1.67)	0.0290
			Mean right SMG dose	1.27 (1.09, 1.48)	0.0024
			Photons	2.38 (1.17, 4.85)	0.0173

n: number of patients with the event/number of patients evaluable

OR: Odds Ratio

CI: Confidence Interval

Dmax: maximum dose in Gy RBE (1.1) to a volume of 0.01 cc

Mean: mean dose in Gy RBE (1.1) to the oral cavity OAR, total parotid glands, left parotid gland, right parotid gland, left submandibular gland, right submandibular gland

V10, 20, 30, 40, 50, 60, 70: volume of oral cavity OAR in cc which receives >= 10, 20, 30, 40, 50, 60, 70 Gy RBE (1.1).

SMG: submandibular gland

RT: radiotherapy

DM: diabetes mellitus
